# Considerations on the State of Medicine in France, from the Time of the Revolution to the Present Period

**Published:** 1819-11

**Authors:** J. B. Regnault

**Affiliations:** Consulting Physician to the King, &c. &c.


FOR THE LONDON MEDICAL AND PHYSICAL JOURNAL.

***Considerations on the State of Medicine in France, from the Time of the Revolution to the present Period.*** **By J. B. REGNAULT, M.D. Consulting Physician to the King, &c. &c.***

ENTHUSIASM may lead to grand and brilliant results in the fine arts; but, in the sciences, its influence is almost always injurious. Yet some writers censure all the productions of the present period, discerning nothing good or beautiful but in those of former times; others, not less exclusive, consider the present only as an enlightened age, seeing in the past nothing but the shades of ignorance and barbarism. The wise man will endeavour to avoid equally those rocks, and to find truth between the two extremes.

The human mind seems to be constantly annoyed by the want of food for its insatiable curiosity. From this irresistible propension towards novelty, as much as from the progressive course of observation, have arisen all the medical theories which have by turn prevailed, from Hippocrates to the existing period.

The instability of those theories, and the obvious obscurities presented by the picture of the healing art to the greater number of physicians, seem at first view to authorize a conclusion, that medicine is the least exact, and the most conjectural, of all the sciences. This notion, which is utterly false, has been favoured by the unjust as well as imprudent criticism that has been conferred on the ancient practice in medicine, and by the extravagant praise given to the labours of the modern schools.

What can persons in general think of a science, which, they say, after having existed two thousand ages, has entirely changed its face within the space of twenty years? In their eyes, medicine can be merely a vain display of rules without a solid basis, and of precepts devoid of utility:—uncertainty is its characteristic; its results, in society, must be either nugatory or deleterious; and the healing art can only be one of those happy frauds, which console the human species by throwing a veil over sad realities.

A popular prejudice has thus been strengthened by the very circumstances which should destroy it; and thus, when medicine daily witnesses the confirmation of those axioms on which it rests, the unfavourable opinion that has been conceived respecting it, has become more fortified from day to day.

It would be easy to show, that this pretended versatility is

* This memoir has been published by Dr. REGNAULT; but a copy of it, with the addition of notes, designating the authors alluded to by the writer, having been transmitted to us by a correspondent at Paris, we are disposed to insert it in this part of our Journal, considering that it will prove instructive, and otherwise useful, to many of our readers.—EDIT.

derived either from the annual and necessary progress of a science gradually enriched by observation and experience, or from a change more or less important in its language or purely theoretical considerations. If we can prove that there does not exist so much difference as may be supposed at a first view, between the method of the earlier practitioners and that of those of the present day, shall we not have contributed to show that medicine, far from being conjectural, is founded on observation;—that it has fixed precepts, certain methods, heroic means, and brilliant conquests, as glorious to the physician as they are useful to human nature;—and that the modifications which have been impressed on it by time, prove the solidity of its principles, instead of unveiling its instability?

Let us for an instant examine the state of medicine in France before the Revolution;—compare what it was then with what it is at the present time, principally directing our attention to the practice of the art;—and we shall see that, with the exception of some innovations, medicine has been always the same, and that its basis has not varied.

We shall not only point out the part which should be attributed to the physicians of the ancient faculties, in the impulse given to medical science at the epoch of the Revolution, but we shall also show with what ardour the professors and the pupils of the new schools have received and favoured that impulse; and we shall designate, in a rapid manner, the results of the labours undertaken in France, during the last thirty years, for the perfection of the healing art.

The subject is immense, and might easily make the matter of a volume: we shall confine ourselves to general considerations, which may perhaps furnish some useful documents to the faithful historian of medicine in the 18th and 19th ages. We could not enter into details in the limits of a memoir; still we may hope to say sufficient to effect the end we have proposed.

In the examination we are about to commence, our only ambition is equally to avoid adulation and satire. Those men who need not fear an investigation of their titles to glory, will applaud our frankness.

We shall not pronounce the name of any living author,—so that the care of making the honourable applications they may please to those who are the objects of them, may be left to the public, always equitable, rather than to the opinion of a single person. In acting thus, we hope to deprive approbation of its insipidity, and censure of its bitterness. Those who disapprove this conduct, will show more vanity than delicacy, more avidity for praise than correctness of sentiment; and we can easily place ourselves above their censure.

If we were disposed to extend our observations to the origin of the healing art, we should state how the experience of Hippocrates, bequeathed by this great man to posterity, became the patrimony of his descendants, and afterwards, that of judicious physicians of all ages and of every enlightened nation. We should see the sublime reveries of antiquity ornament, or rather overcharge, the sentences of the divine sage, and alter even the sense of them, but never cause them to be forgotten. We should see the Greeks, the Romans, the Arabians, the Italians, the French, the Germans, the English, and the Spaniards, gradually add some lines to the immortal tablets of the Prince of Physicians;—insensibly augment the treasure of experience;—and drive to the last extremities the sectaries who have wished to proscribe the memory of a great man, whose name alone is for them a refutation, and even a satire.

Many errors, many sterile hypotheses, have without doubt been mingled with the doctrine of Hippocrates; but this doctrine has remained unshaken amidst all the systematic tinsel with which it has been charged: it has been cherished by all those physicians who have especially devoted themselves to the improvement of the art. The most wise, however, have not disdained the fruits of recent observation; they have profited, to the great advantage of human nature, by the labours of their contemporaries, taking care to disengage them from the incoherent, or at least always useless, explanations with which they have been disfigured. Indeed, when the dogmas of Galenism, of medical chemistry, and of Boerhaavisme, absorbed the whole attention of vulgar physicians, a few judicious observers, docile to the voice of nature and of truth, listened only to the precepts of experience, and complacently smiled at the declamations of the fanatic admirers of the predominant systems.

It is not, then, at the present period only that the happy disposition to confine ourselves to facts as they are presented to us by nature, has been assumed: it is not at the present period only that men of cool understanding, equally disdaining solidism and humorism, mechanism and Brownism, have appeared. Physicians have existed in all ages, who, after having, by the aid of analysis, so natural to the human mind, deduced from the aspect of the patient all the circumstances dependant on idiosyncracy, have been content to study the symptoms, to investigate the seat, of the disease, and thence to determine by analogy the necessity of employing the particular means of which experience, the only judge in this important point, had demonstrated the efficacy.

The progress of anatomy, without producing any change in these principles, has given a more assured mode of conduct to those men of superior talents who follow this excellent method,

If anatomical knowledge were formerly less generally diffused than at the present time, it was not because its utility was not understood, but because an unusual degree of courage was necessary to lead a person to shut himself up in a frightful cell, infected with the remains of dead bodies kept for a period of two months;—it was because it was necessary for a person to expose himself to the almost inevitable dangers of a disease often mortal, in receiving a sort of instruction, of which all men of genius were nevertheless ardently desirous. The writings of Lecat, however, at length inspired them with all the ardour necessary to surmount the numberless obstacles to which they had been exposed by a defective mode of education.

The memory of A. Petit deserves also to be recalled: no less able as a professor than as a practitioner, he could deprive anatomy of all its dullness, and give to his lectures a charm but little known.

Pathological anatomy was studied in the writings of Bonnet (Bonetus), Morgagni, Lieutand, and in the traces of Desbois of Rochefort;—physiology and comparative anatomy, in the immortal work of Haller, and in the course of the eloquent Vicq-d'Azyr,—of Vicq-d'Azyr, whose elocution was so refined, whose knowledge was so profound, and whose views were so extended;—of Vicq-d'Azyr, too much neglected at the present day, and who should nevertheless be justly considered as the founder of physiological instruction in the school of Paris. The friend of truth must read with pleasure the fine eulogium of this great man, traced by the pen of the eloquent author of the *Nosologie Naturelle.** Why has not every pupil rendered to his master the justice they owe to him? This reproach cannot fall on the learned editor† of the works of the Perpetual Secretary of the Royal Society of Medicine.

A physician,‡ equally claimed by the two branches of the art, and who long since commenced the most honourable career as a professor and as a practitioner, conceived and executed the first project of a plan of medical anatomy. This work is not the least of the titles to glory which he has merited by his excellent treatises on several points of the practice of medicine. Called to the first place near a monarch who is an enlightened appreciator of true merit, this venerable dean of the physicians of France worthily represents two ages of a science, to which he has attached his name in a brilliant manner.

At the epoch to which we allude, Sauvages served as a guide in the study of the nomenclature of diseases, and of their different species and varieties. The imperfections of his nosology were not dissimulated; but these faults, counterbalanced by

* M. Alibert. † M. Moreau de la Sarth. ‡ M. Portal.

great advantages, were not in any way dangerous. Cullen, however, reduced the table of diseases to more just proportions, and diminished the number of species, evidently too multiplied by the learned professor of Montpellier.

The work of Gaubius was, it is true, the only manual we had then on the knowledge of diseases; but, if it has become antiquated, it has not been replaced by any other worthy of being compared to it.

The meditation of the writings of Baillou, Sydenham, Baglivi, Hoffman, Boërhaave, and Van Swieten, the perusal of which is now too much neglected;—the Clinical Treatise of Desbois of Rochefort, the study of his Materia Medica and his excellent practical Lectures; completed the course of the young physician, really jealous of worthily qualifying himself for the exercise of the most honourable of professions.

Whilst Louis, Sabatier, Peyrhill, Desault, and Baudelocque, enlightened in their courses a rising generation ardently desirous of the acquisition of knowledge, the immortal Academy of Surgery dictated to European surgery the laws which are still respected; and left, in the collection of its memoirs, an heritage that will be for ever celebrated.

Macquer, Darcet, Lavoisier, Fourcroy, and Guyton-de-Morveau, prepared, by their labours and discoveries, the success of the chemists who have replaced them. We should also remark, that the successors of those great men have been just and grateful to their masters; and we feel pleasure in rendering this tribute to their honourable conduct. Why can we not say as much of all physicians!

Baume, notwithstanding his opposition to the new chemical nomenclature, will ever be an authority in pharmacy: his work is still the manual of all the pharmaceutists of Europe.

After having thus very concisely indicated the course of medical study followed before the Revolution, let us see what were the practical opinions then generally adopted.

Many persons believe, or rather feign to believe, that, thirty years since, to bleed and purge in a purely empirical manner, formed the whole code of a practical physician. It is thus that ignorance and dishonesty assiduously conspire against truth. Nothing is more easy than to reply to those who thus contemn the ancient practice of medicine.

The doctrine of fevers was, it must be acknowledged, founded solely on their symptoms: these maladies were considered as general affections of the system, especially by the practitioner who was an enemy to all hypothesis; but matters did not therefore take a worse course; for a too-exclusive theory had not yet taught physicians to see every where nothing but excess of power or of debility. A hard and frequent, or concentrated, pulse; intense heat of the skin; dryness and redness of the tongue; determined the true practitioner to employ immediately one or two bleedings, according to the strength of the patient: he knew, from Sydenham, Stoll, Van Swieten, and, above all, from observation, that a too-heating mode of treatment hastens, in such cases, the development of the symptoms to which the appellation putrid was then given, though this denomination did not prove hurtful in any way to the determined conduct of the physician. Besides, Selle soon produced his Pyretologie, which was received in France with eagerness, and of which attempts have too soon been made to lessen the value.

They were not then so prodigal of purgatives as some persons now assert: to be convinced of it, it is only necessary to read what Desbois said respecting the cases in which they are indicated. Stoll, in his immortal works, who was in the hands of every pupil and every practitioner,—Stoll forcibly pointed out the ill consequences of the abuse of those means so beneficial in certain circumstances, and so deleterious in many others. Many physicians had, without doubt, a sort of predilection for evacuant remedies: but are there not any of this kind now existing? The human mind but rarely keeps a just medium: and it is only a few physicians who have neither exclusive love nor hate for the different agents the three kingdoms of nature give to their disposal.

Emetics were not so lavishly employed as they have since been: physicians knew how necessary it was to be reserved in the use of these violent measures; and they had not yet arrived at the practice of administering them indiscriminately at the commencement of all diseases. They knew that, in some cases where this heroic remedy appears to be contra-indicated, it will however act with great efficacy; but its exhibition in these uncommon cases was confined to the more experienced practitioner; whilst now, the most contemptible *curer* prescribes it without distinction in all diseases, with an assurance that would excite astonishment, if we did not in the end recognize ignorance proceeding blindly through every difficulty, under the authority of others.

Vesicatories were often employed, less as evacuants than as powerful means of derivation and irritation.

Alphonso Lerois had already taught all the advantage that might be deduced from the application of leeches, especially in convulsions affecting infants.

Acute inflammations, the history of which has been so brilliantly elucidated by the progress of pathological anatomy since Morgagni, were hardly less well understood than at the present time. Something, however, yet remained to be done for their methodic classification; but their chronic state had already fixed the attention of some celebrated practitioners. The researches of Stoll respecting latent inflammations are well known,—researches that have served as a model to all the subsequent labours to perfect the diagnosis of slow and chronic inflammations.

It would occupy us too long to run over the whole nosological table, and show how, at the epoch to which we allude, all the diseases were considered, and especially in what manner they were treated; let it suffice to say, that the excellent practical rules of Baillou, Sydenham, Baglivi, Stoll, Riviere, Bordeu, Fouquet, Lorry, Quesnay, Bouvard, and Barthez, directed young physicians in the treatment of their patients,—but that experience was always consulted respecting the administration of the curative measures.

If we were to dwell on the picture of medicine in France before the Revolution, we should have to show that then, as at present, Paris was the centre of acquirements in medicine; that, at the formation of the new faculties, the professors brought forward traditions which they had collected in the ancient schools; and, conformable to justice, we should add, that they have themselves powerfully contributed to the ulterior progress of the healing art.

Descriptive anatomy, taught with so much care by Desault, has given rise to many treatises,* more complete than those of Winslow and Sabatier; yet these new works have not added much to those of these celebrated anatomists, who had the sense not to surcharge the memory of students with details from which surgeons could derive no utility.

Comparative anatomy has made great progress, and furnished some highly valuable illustrations to physiology. The honour of this is particularly due to the French. A treatise on this science, published by the first of our naturalists,† has obtained the approbation of the whole of the enlightened part of Europe, and has produced the most warm and happy emulation in the laborious anatomists of Germany. Many parts of natural history have been treated with great success by the coadjutors‡ of the illustrious philosopher we have just designated; and in this respect France still serves as a model to other nations.

The deep and comprehensive physiological views of Haller, Bordeu, Grimaud, Barthez, Vicq-d'Azyr, and Dumas, collected and appreciated by Bichât, with which he was inspired by an

---

* Those of MM. Bayle, Cloquet, and Marjolin.

† M. G. Cuvier.

‡ MM. Geoffroy de St. Hilaire, de la Marck, and F. G. Cuvier.

able master,* excited in him a generous ardour; and, soon becoming himself rich in a great number of facts, he joined them to all those which had been observed by the great men we have just designated; and from that time the name of general anatomy was given to a part of anatomy which had not previously formed a body of doctrine.

We should be ill understood, if it were thought that we wished to sully the justly-acquired glory with which a grateful posterity has gratified the memory of Bichât; but we cannot refrain from saying, that he did not act justly towards a great number of other authors, and particularly towards Haller; and, above all, Barthez, of whom he has hardly said a single word.

Amongst the numerous experiments made on animals, there are but few which have given rise to very advantageous results; and it is much to be desired, that the study of man, in the state of health and in that of disease, should not be neglected, to seek in the viscera of animals what will there never be found.

Nothing appears to us more remarkable, than the application made by Cabanis of modern philosophy to medicine. This immortal author should be reckoned amongst the number of those who have the most contributed to the advancement of medical philosophy: his work† is a fine monument, raised in honour of the sciences in the nineteenth century.

[*To be continued.*]

